# Propensity score matching as an effective strategy for biomarker cohort design and omics data analysis

**DOI:** 10.1371/journal.pone.0302109

**Published:** 2024-05-02

**Authors:** Masaki Maekawa, Atsushi Tanaka, Makiko Ogawa, Michael H. Roehrl

**Affiliations:** 1 Department of Pathology, Beth Israel Deaconess Medical Center, Boston, MA, United States of America; 2 Harvard Medical School, Boston, MA, United States of America; 3 Memorial Sloan Kettering Cancer Center, New York, NY, United States of America; University Magna Graecia of Catanzaro, ITALY

## Abstract

**Background:**

Analysis of omics data that contain multidimensional biological and clinical information can be complex and make it difficult to deduce significance of specific biomarker factors.

**Methods:**

We explored the utility of propensity score matching (PSM), a statistical technique for minimizing confounding factors and simplifying the examination of specific factors. We tested two datasets generated from cohorts of colorectal cancer (CRC) patients, one comprised of immunohistochemical analysis of 12 protein markers in 544 CRC tissues and another consisting of RNA-seq profiles of 163 CRC cases. We examined the efficiency of PSM by comparing pre- and post-PSM analytical results.

**Results:**

Unlike conventional analysis which typically compares randomized cohorts of cancer and normal tissues, PSM enabled direct comparison between patient characteristics uncovering new prognostic biomarkers. By creating optimally matched groups to minimize confounding effects, our study demonstrates that PSM enables robust extraction of significant biomarkers while requiring fewer cancer cases and smaller overall patient cohorts.

**Conclusion:**

PSM may emerge as an efficient and cost-effective strategy for multiomic data analysis and clinical trial design for biomarker discovery.

## Background

Omics data analysis has become increasingly popular in biomedical research and precision medicine. Due to advances in sequencing and other deep molecular profiling methods and infrastructure, a wealth of multiomic data, spanning from the genome, transcriptome, proteome to the metabolome, have been generated. Omics datasets are complex and associated with multiple biological and clinical parameters. There is currently no standard method for parsing through omics data to extract significant specific biomarkers and separating them from a multitude of possibly confounding variables. Typically, very large randomized patient cohorts are considered to be needed in disease biomarker discovery for addressing the confounding variable problem.

Propensity score matching (PSM) is a statistical matching technique that attempts to deduce the effect of a single specific factor by reducing bias due to confounding variables [1]. In past studies, the assignment of a single specific factor to a subject was not random, and there were many confounding variables between the group with the specific factor and the control group without the factor. The propensity score expresses quantitatively “how likely each case is to have the specific factor.” The estimated propensity score *e*(*x_i_*) of each case can be calculated based on its background, *i* (*i* = 1,2,…,*N*), which could be a confounding variable. Paired cases with most similar propensity scores can be extracted from each group with or without the single specific factor [2–5]. This process of homogenizing confounding factors is called PSM, and it enables to obtain extracted groups and control groups which have a difference only in the single specific biomarker to be examined.

Although PSM is a powerful statistical method, it has not been implemented into routine omics data analysis for biomarker discovery. In this study, we tested PSM in two colorectal cancer datasets to investigate its efficiency in omics analysis. One dataset was derived from immunohistochemical (IHC) protein expression of 12 protein markers in a cohort of 544 colorectal cancer (CRC) patients [6, 7]. The second dataset was obtained from The Cancer Genome Atlas database (TCGA) and contained total RNA-sequencing profiling of 163 CRC patients [8]. We clustered the cancer cases into good or poor prognosis groups based on patient survival information, and the groups were compared to uncover prognostic protein or transcript biomarkers. We then compared the results before and after PSM implementation to evaluate the efficiency of PSM for omics data analyses and confounding variable-avoiding biomarker discovery.

## Methods

The study used retrospective archives samples and was classified by the institution as minimal risk and with waiver of consent (BIDMC IRB 2023P000933).

### Dataset of proteomic markers of CRC

This dataset was derived from proteomic expression profiles of 544 surgically resected CRC tissue obtained from the pathology archives of MSKCC. Clinicopathological parameters, including patient age, gender, tumor location, TNM stage, mismatch repair (MMR) status, histology, tumor differentiation, lymphovascular invasion, and perineural invasion were retrieved from medical records. This cohort comprised 367 patients who survived more than three years without recurrence (good outcome group), and 60 patients who had recurrence within three years (poor outcome group). The others did not have sufficient survival information due to short follow-up periods.

Twelve potential prognostic biomarkers (NNMT, GALNT6, SLC3A2, SLC7A5, IGF2BP3, MCM6, SERPIN B5, STAT1, NAMPT, P4HA1, DDX21, and LTBP2) were chosen based on preliminary proteomic findings from our laboratory [6, 7]. Expression levels of these proteins were determined by immunohistochemical (IHC) staining of tissue microarrays. The tissue microarrays were constructed from 544 formalin fixed and paraffin embedded CRC tissue specimens. Tissue sections were incubated with polyclonal antibodies specific to each protein marker (see S1 Table for details on antibodies used) and visualized with Bond Polymer Refine Detection (Leica). IHC staining scores, which correspond to protein expression levels in cancer tissue, were determined independently by two pathologists without knowledge of the patients’ clinical information.

For seven markers, IHC staining intensity of individual tumor cells was determined and assigned intensities of 0, 1+, 2+, or 3+, and the total weighted IHC score of a sample slide was calculated by multiplying the expression intensity of individual tumor areas (score 0–3) by their relative contribution (0–100%) to total tumor area. The total weighted IHC scores thereby had a range of 0–300 for these protein biomarkers. For NNMT, GALNT6, or MCM6, each tissue section was scored by counting the number of cancer cells staining positively for the protein biomarker (staining intensity ≥1+) relative to the total number of evaluated cancer cells. A minimum of 500 cancer cells were evaluated per tissue sample, and the IHC scores had a range of 0–100. For DDX21 or LTBP2, IHC stains were scored binomially as negative or positive (score 0 or 1+).

### Random forest analysis

IHC scorings were compared between the good and poor prognosis groups. A random forest analysis was performed to detect prognostic IHC markers using the R (version 4.2) packages “randomForestSRC” (version 3.3.2) and “ggRandomForests” (version 2.2.1) downloaded from *cran*.*rstudio*.*com*. The analysis included recurrence-free survival data, clinicopathological features, and IHC scorings. The number of trees used was set to 100,000. Minimal depth value and variable importance were utilized to detect significant prognostic factors. Additionally, the significant prognostic IHCs which were selected by random forest analysis were compared to the results of the original comparisons.

### Statistical comparison

Clinicopathological values were compared using a Student t-test or a chi-squared test if the values were binary. The comparisons of IHC scorings were also conducted using a Student t-test or a chi-squared test.

### Dataset of CRC transcriptome

The original RNA-seq dataset used in this study is available using the “GDCquery” function in the package “TCGAbiolinks” (version 2.18.0) downloaded from *bioconductor*.*org* (R version 4.2). The “project” names used are “TCGA-COAD” and “TCGA-READ”. The “data.category” name used is “Transcriptome Profiling” and the “data.type” used is “Gene Expression Quantification” for RNA-seq data. The “data.category” name used is “Clinical” for clinical information.

This dataset contains mRNA sequencing counts for 60,660 genes from 163 CRC patients [8]. The data were obtained from TCGA-COAD and TCGA-READ using the Bioconductor package in R [9, 10]. In this CRC cohort, 130 patients survived more than three years after sample collection and were considered in the good prognosis group, and 33 patients died within three years and were considered in the poor prognosis group. The dataset included clinicopathological information such as age, gender, TNM stage, MMR status, histology, residual tumor status, venous invasion, lymphovascular invasion, and perineural invasion.

### Differential gene expression analysis

Differential expression analysis was performed to identify genes that were significantly associated with outcome separating good and poor prognosis groups. Differential transcriptome expression analysis of was conducted using the R (version 4.2) packages “limma” (version 3.56.2) and “edgeR” (version 3.42.4) downloaded from *bioconductor*.*org* [11–13]. Transcripts with counts per million (CPM) <15 were filtered out in order to exclude low-expression genes, and the dataset was normalized. The dispersion of gene expression values was estimated [14], and statistical tests of fold change were computed to compare the poor prognosis group to the good prognosis group. Gene transcript changes with a false discovery rate (FDR) less than 0.05 were considered significant.

### Propensity score matching (PSM)

**Fig 1:**
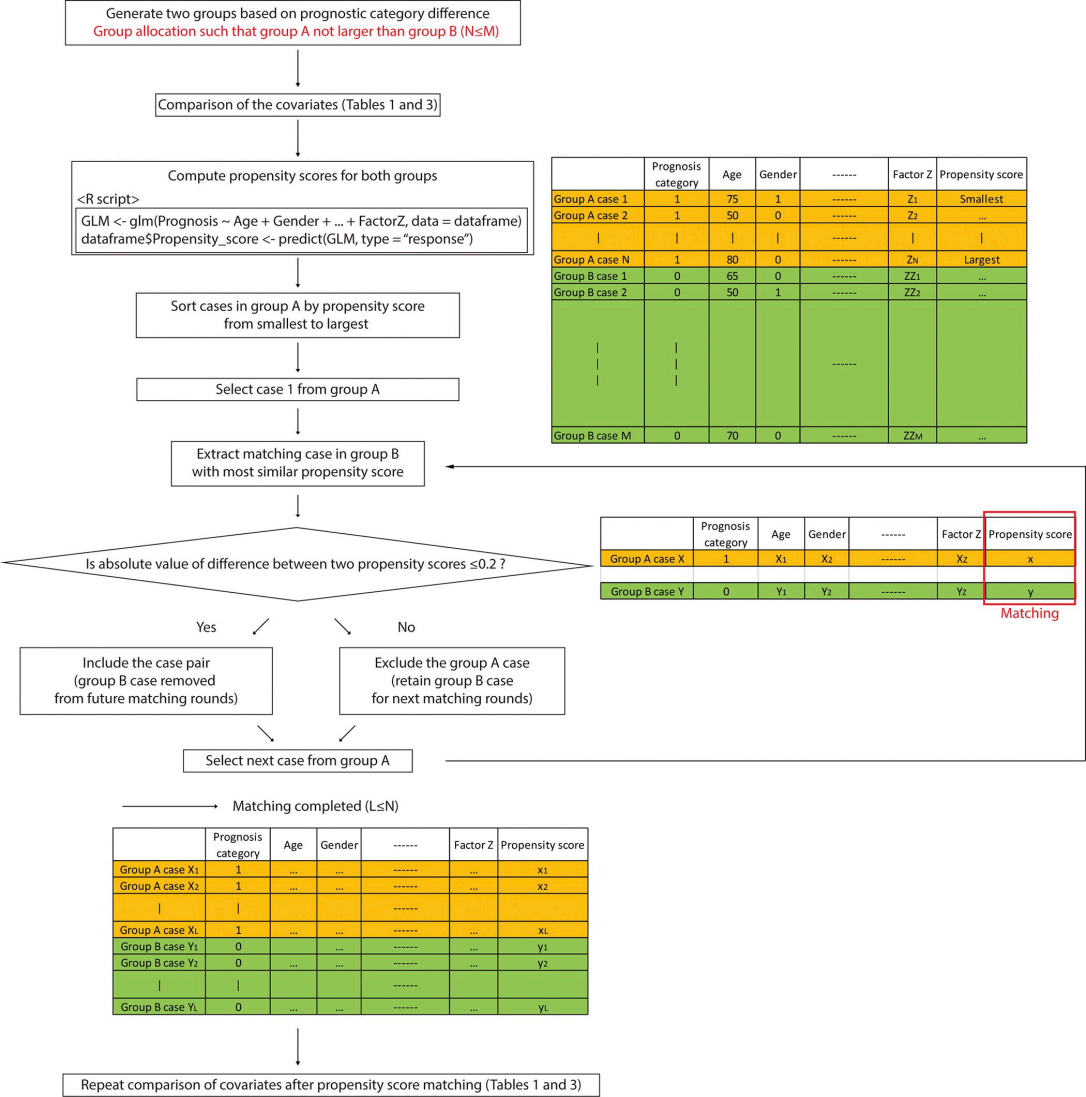
Flow chart of propensity score matching in this study. It is crucial that the number of cases in group A is not larger than in group B. In this study, group A meant the good prognosis group, and group B meant the poor prognosis group in both datasets.

A flow chart of how PSM was performed in this study is shown in Fig 1. Estimated propensity scores of each case *e*(*X_i_*) were calculated using linear logistic regression:

$$\ln\frac{e(x_{i})}{1-e(x_{i})}=\ln\frac{P_{r}(Z_{i}=1|x_{i})}{1-P_{r}(Z_{i}=1|x_{i})}=\alpha +\beta^{T}x_{i}$$

$$e(x_{i})=\mathrm{Pr}\left(Z_{i}=1\right|x_{i})$$

$$e(X_{i})=b_{0}+b_{1}X_{1}+b_{2}X_{2}+\cdots +b_{i}X_{i}$$

*b*_0_: the intercept

*b_i_*: the regression coefficient

*x_i_*: the variables of each case

*X_i_*: the specific variable to investigate and covariates

*Z_i_* = 1: case in a poor prognosis group

*Z_i_* = 0: case in a good prognosis group

The propensity score of each case was computed from the collected clinicopathological features using the “glm” function (generalized linear model) and the “predict” function, both of which are part of the basic R package “stats” (version 4.4.0).

GLM<−glm(Prognosis∼Age+Gender+⋯,data=dataframe)

dataframe$PropensityScore<−predict(GLM,type="response")

Cases in the group with the smaller number of patients (in this study, the poor prognosis group) were sorted by their propensity scores. The sorting was done from smallest to largest propensity score, and, for each case, a paired case with most similar propensity score was selected from the good prognosis group. If the absolute value of difference between two propensity scores was less than 0.2, the case pair was included and the matched case removed from future matching rounds. If the absolute value of difference was greater than 0.2, the original case was excluded and the matched case retained for next matching rounds. This process resulted in the creation of two extracted groups that had the same number of cases without duplicates, and the number of cases was smaller than the original dataset.

## Results

### Prognostic proteomic biomarkers of CRC

**Fig 2:**
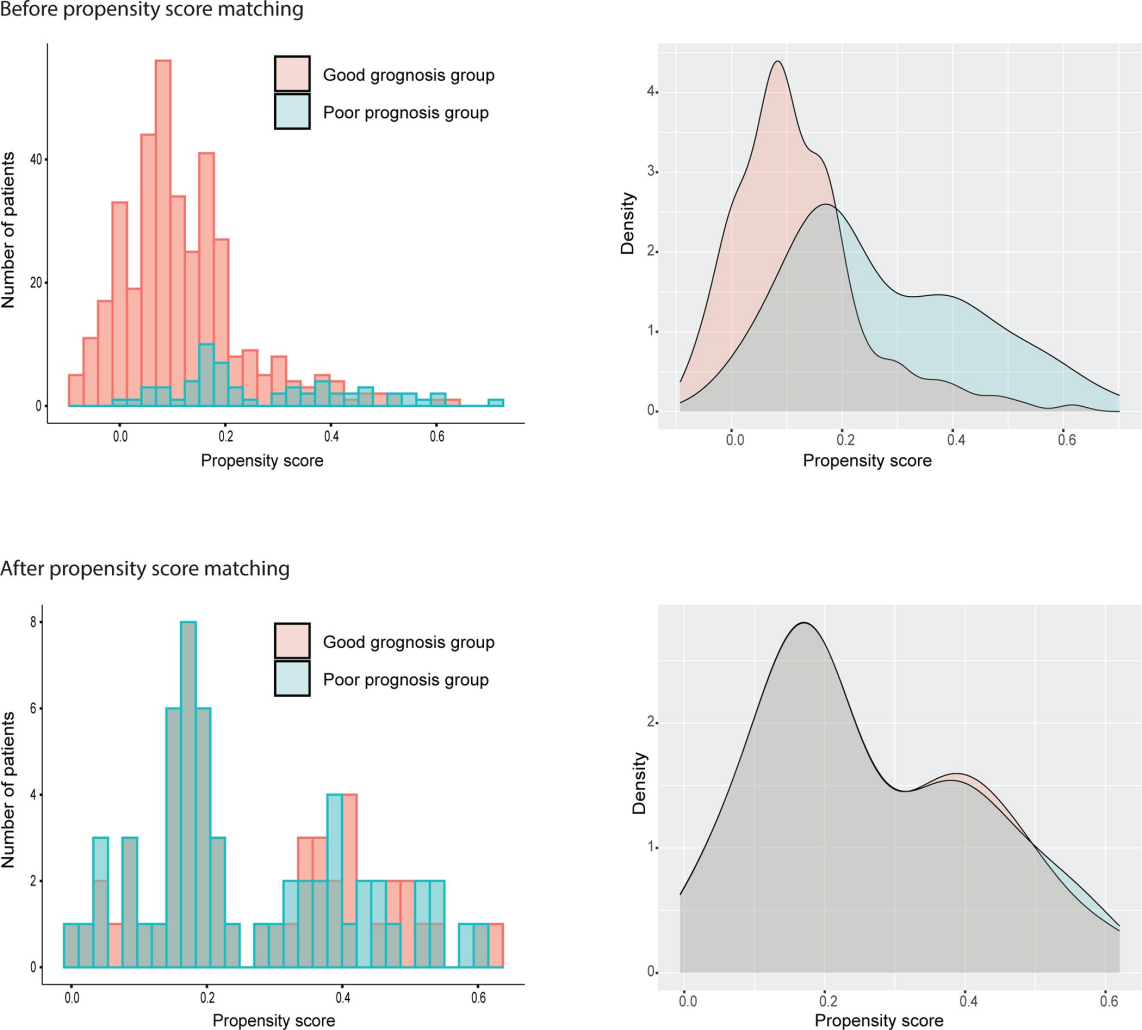
Distribution of propensity scores of the IHC CRC dataset.

The proteomic marker dataset of CRC cohort consisted of 367 cases in the good prognosis group and 60 cases in the poor prognosis group (Table 1). Propensity scores were computed for each case, and the resulting distributions of the propensity scores are shown in histograms and density plots (Fig 2). After PSM, group differences of confounding factors, such as age, gender, TNM stage, lymphatic invasion positivity, and perineural invasion positivity, were eliminated. Two paired groups of 58 cases each were created after PSM, a significant reduction in case numbers in both the good and poor prognosis groups when compared to the original data before PSM (Table 1).

**Table 1:** Clinicopathological features of the proteomic CRC cohort before and after PSM.

	Before PSM			After PSM		
	Good prognosis (N = 367)	Poor prognosis (N = 60)	p-value	Good prognosis (N = 58)	Poor prognosis (N = 58)	p-value
Age (mean)	64.0	56.4	**< 0.001**	55.3	56.8	0.604
Gender			**0.01889**			0.3426
Male	175	39		32	38	
Female	192	21		26	20	
Location			0.1291			0.3426
Right	183	23		20	23	
Left	184	37		38	35	
TNM staging (mean)	1.72	2.35	**< 0.001**	2.31	2.29	0.9087
Mismatch repair status			0.2982			0.5995
Present	87	10		7	10	
Absent	280	50		51	48	
Histology			0.8768			1
Mucinous	25	5		5	5	
Not mucinous	342	55		53	53	
Tumor differentiation (mean)	2.02	2.08	0.2159	2.07	2.09	0.8171
Lymphatic invasion			**< 0.001**			1
Positive	64	27		26	25	
Negative	303	33		32	33	
Perineural invasion			**< 0.001**			1
Positive	18	15		13	13	
Negative	349	45		45	45	

Bold: p <0.05, statistically significant

Comparisons of protein marker expression as measured by IHC scores between the good and poor prognosis groups before and after PSM are shown in Table 2. In the analysis before PSM, three protein biomarkers (SLC7A5, SLC3A2, and STAT1) showed statistically significant differences between the good and poor prognosis groups. However, only STAT1 showed significant difference between the two groups after cohorts were minimized for confounding variables by PSM.

**Table 2:** Comparison of protein marker expression between groups before and after PSM.

	Before PSM			After PSM		
	Good prognosis (N = 367)	Poor prognosis (N = 60)	p-value	Good prognosis (N = 58)	Poor prognosis (N = 58)	p-value
NNMT	35.4	37.1	0.5295	38.1	37.3	0.8212
GALNT6	54.6	50.5	0.349	47.8	51	0.5961
SLC7A5	84.1	60.7	**0.005109**	72	62.7	0.3822
SLC3A2	88.1	60	**0.005121**	60.5	62.1	0.9039
IGF2BP3	97.3	96.6	0.9275	106	96.9	0.4854
MCM6	13.8	19.3	0.1322	19.2	18.9	0.9473
SERPIN B5	86.7	70.2	0.06452	77.6	70.9	0.555
STAT1	57.1	25.3	**< 0.001**	59.1	26.2	**0.002791**
NAMPT	91.5	89.8	0.8424	100.2	89.1	0.356
P4HA1	95.8	108.2	0.1576	115.5	108.1	0.5195
LTBP2 (cytoplasm) positive	136	26	0.4323	18	26	0.1804
LTBP2 (stroma) positive	79	12	0.9223	14	11	0.6515
DDX21	81	9	0.2827	10	8	0.7976

Bold: p <0.05, statistically significant

**Fig 3:**
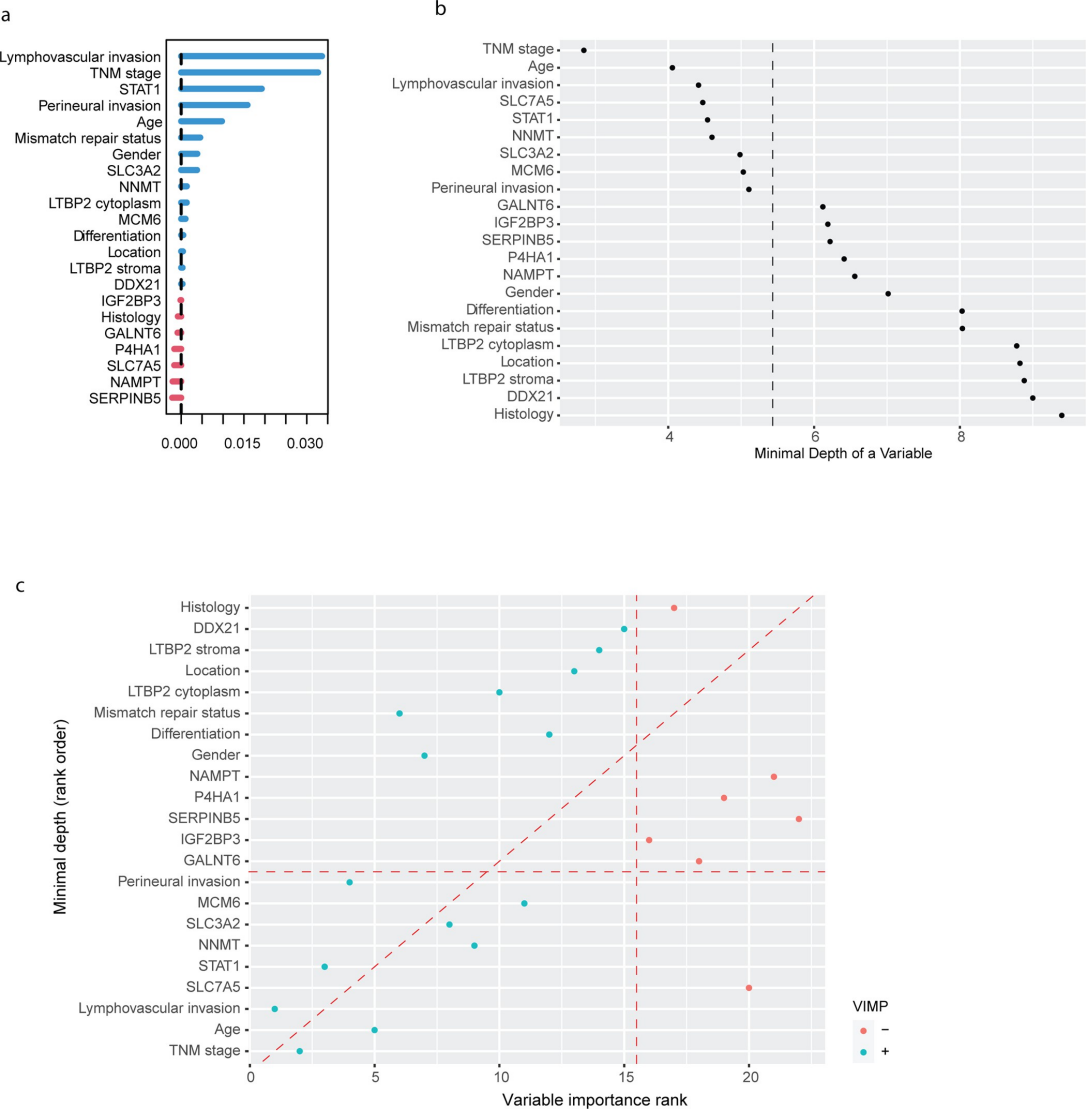
Random forest rankings of prognostic factors in the CRC proteomic marker dataset. **(A) Ranking of variable importance (VIMP).** The blue bars represent positive values of VIMP, indicating that the corresponding factor is positively associated with prognostic prediction. While the red bars represent negative values of VIMP, indicating that the factor is negatively associated with prognostic prediction. **(B) Ranking of minimal depth.** The small minimal depth indicates that the factor plays an important role in prognostic prediction. The vertical dashed line indicates the minimal depth threshold where smaller minimal depth values indicate higher importance and larger indicate lower importance as calculated by the “gg_minimal_depth” function of the “ggRandomForests” R package (version 4.7–1.1). **(C) The combination of variable importance (VIMP) and minimal depth.** The blue dots represent positive values of VIMP, while red dots represent negative values of VIMP. The threshold represented by the vertical red dashed line indicates VIMP = 0. The threshold represented by the horizontal red dashed line is equal to (B).

To validate the above findings, we also conducted random forest analyses on the dataset to identify significant prognostic factors (Fig 3). In addition to clinicopathological features, several markers such as STAT1, NNMT, SLC3A2, and MCM6 were found to be significant prognostic factors based on both minimal depth and variable importance rankings. Except for clinicopathological features such as lymphatic invasion and TNM stage, STAT1 was the most significant prognostic factor based on both types of rankings from random forest analysis.

### Prognostic transcriptomic biomarkers of CRC

**Fig 4:**
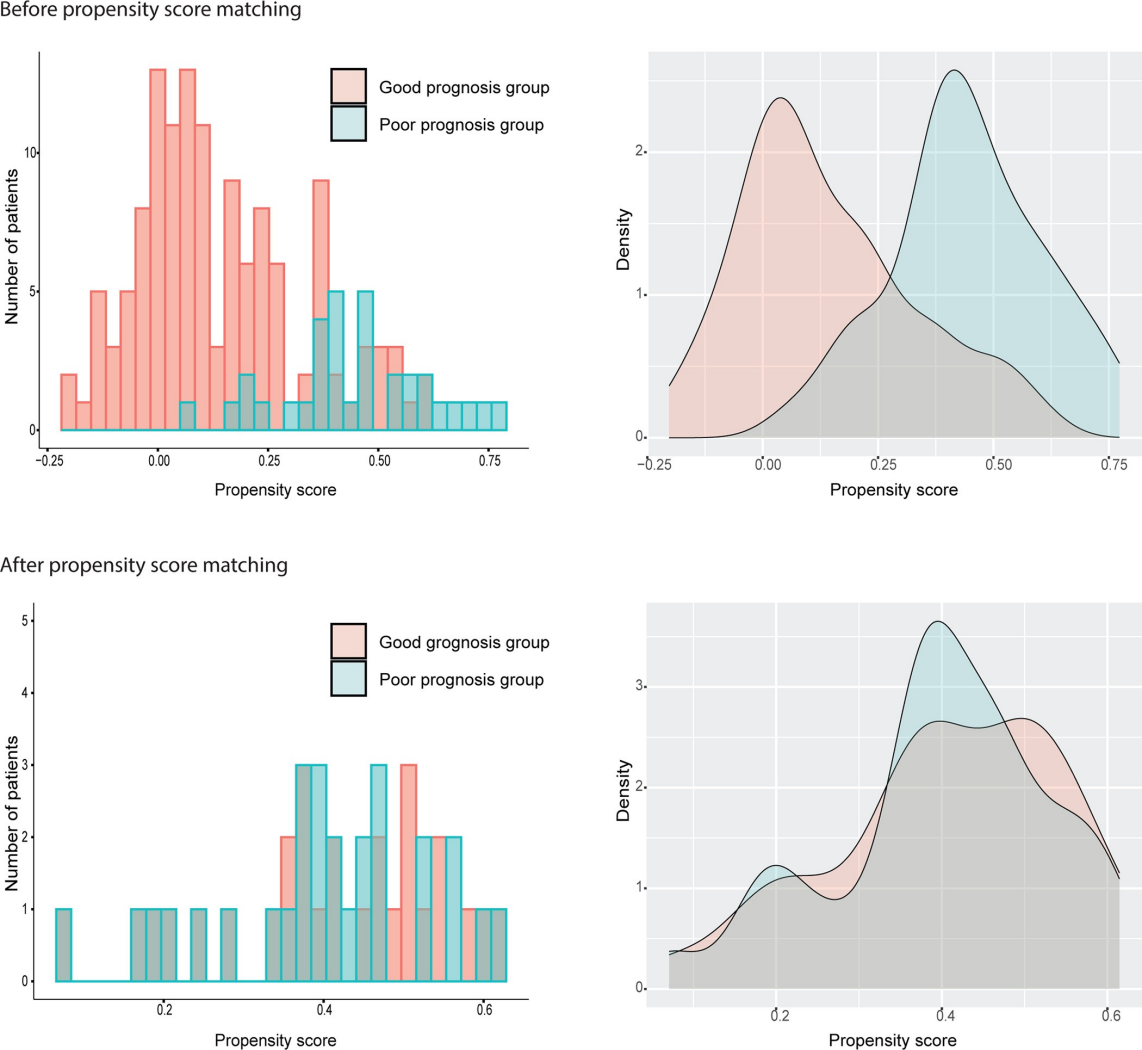
Distribution of propensity scores of the RNA–seq CRC dataset.

The TCGA’s CRC RNA-seq dataset consisted of 130 cases in the good prognosis group and 33 cases in the poor prognosis group (Table 3). The propensity score of each case was calculated, and the distributions of these scores were shown in histograms and density plots (Fig 4). Before PSM, the good and poor prognosis groups were significantly impacted by six confounding clinicopathological features, including TNM staging, mucinous histology, residual tumor status, venous invasion, lymphatic invasion, and perineural invasion. These confounding features were successfully eliminated by PSM, resulting in two paired groups of 28 cases each with no significant differentiating clinical factors (Table 3).

**Table 3:** Clinicopathological features of the CRC RNA–seq dataset before and after PSM.

	Before PSM			After PSM		
	Good prognosis (N = 130)	Poor prognosis (N = 33)	p-value	Good prognosis (N = 28)	Poor prognosis (N = 28)	p-value
Age (mean)	65.3	68.3	0.2154	69.3	67.4	0.5481
Gender			0.976			1
Male	72	19		17	16	
Female	58	14		11	12	
TNM staging (mean)	2.23	3.15	**< 0.001**	3.0	3.0	1
Mismatch repair loss			0.2156			-
Present	10	0		0	0	
Absent/unknown	120	33		28	28	
Histology			**0.0096**			1
Mucinous	9	8		6	7	
Not mucinous	121	25		22	21	
Residual tumor status			**< 0.001**			0.7748
No	88	10		8	10	
Other/unknown	42	23		20	18	
Venous invasion			**0.010**			1
Positive	22	13		10	9	
Negative/unknown	108	20		18	19	
Lymphatic invasion			**0.013**			0.7844
Positive	32	16		10	12	
Negative/unknown	98	17		18	16	
Perineural invasion			**0.033**			1
Positive	9	7		4	3	
Negative/unknown	121	26		24	25	

Bold: p <0.05, statistically significant

Without PSM implementation, total mRNA sequencing of the original 130 good prognosis cases and 33 poor prognosis cases yielded 13,294 genes (21.9% of all) with counts per million (CPM) greater than 15. Differential gene expression analysis identified 402 genes with false discovery rate less than 0.05, i.e., significantly differentially expressed between the good and poor prognosis cases. They included 373 upregulated and 29 downregulated genes associated with poor prognosis.

After PSM and reducing the cases to only 28 in each group, differential expression analysis identified 12,460 genes (20.5% of all) with CPM greater than 15. 122 differential genes were observed with false discovery rate less than 0.05. The 122 significant genes included 109 upregulated and 13 downregulated genes associated with poor prognosis.

**Fig 5:**
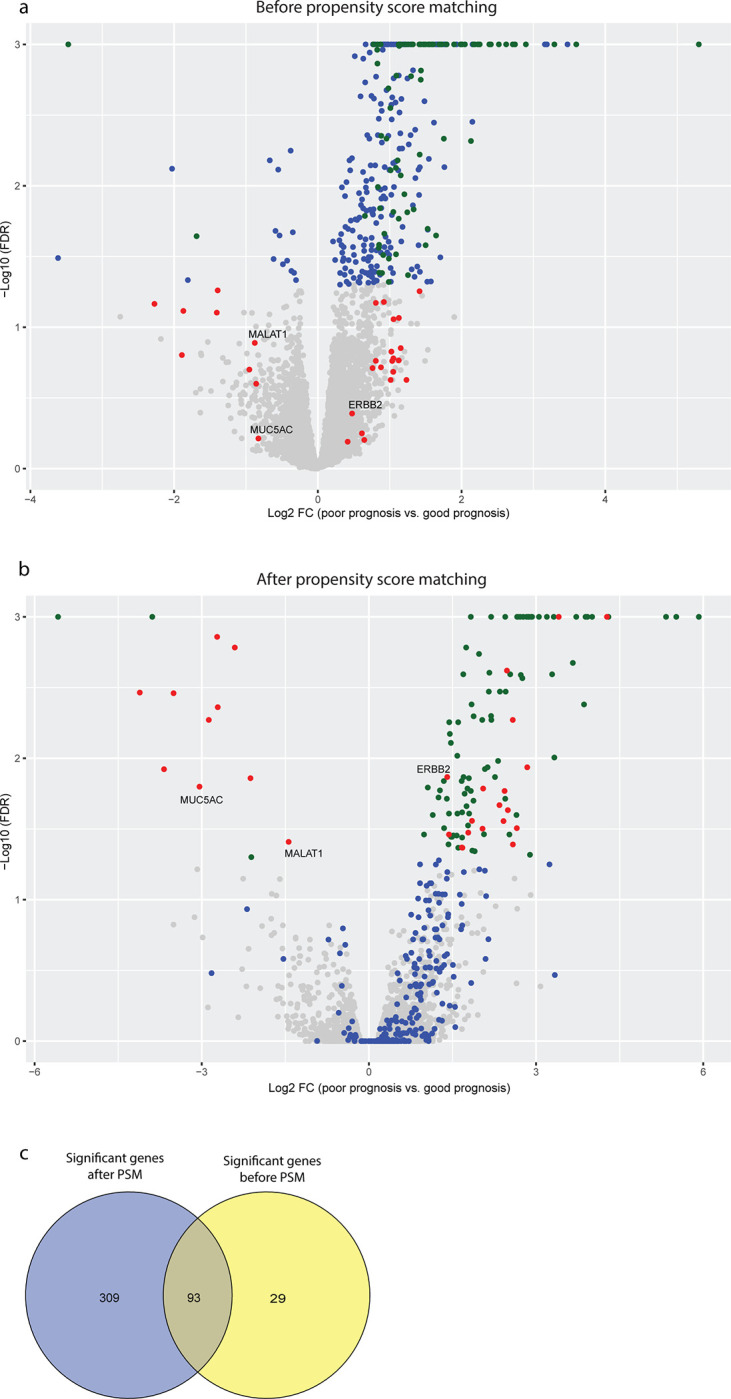
(A–B) RNA–seq volcano plot comparing good prognosis group vs. poor prognosis group. Green dots (N = 93) represent genes that are significant in both pre–and post–PSM comparison between the good–and poor–prognosis groups. Blue dots (N = 217) represent genes that are significant only in the pre–PSM comparison between the good–and poor–prognosis groups. Red dots (N = 29) represent genes that are significant only in the post–PSM comparison between the good–and poor–prognosis groups. Grey dots (N = 12,121) represent genes that did not show significant differences. **(C) The Venn diagram of significant genes before and after PSM.** The blue circle represents before PSM, and the yellow represents after PSM.

Comparing the results before and after PSM, 29 significantly differentially expressed genes were only identified after PSM, while 93 significant genes were identified both without and with PSM analysis (Fig 5). The 29 newly uncovered CRC outcome-differential genes are listed in Table 4. Additionally, the 93 genes selected in common across both comparisons are provided as S2 Table.

**Table 4:** Genes significantly associated with CRC prognosis uncovered only by PSM.

Gene	Description	Evidence as CRC biomarker?
MTND1P23	MT-ND1 pseudogene 23	[15]
AFAP1-AS1	AFAP1 antisense RNA 1	[16]
LEFTY2	left-right determination factor 2	
SCARA5	scavenger receptor class A member 5	[17]
CADM3	cell adhesion molecule 3	[18]
MALRD1	MAM and LDL receptor class A domain containing 1	
ABCA8	ATP binding cassette subfamily A member 8	
ANPEP	alanyl aminopeptidase, membrane	[19]
GREM2	gremlin 2, DAN family BMP antagonist	
CHRDL1	chordin like 1	
CILP	cartilage intermediate layer protein	
STMN2	stathmin 2	
LYVE1	lymphatic vessel endothelial hyaluronan receptor 1	[20]
RGMA	repulsive guidance molecule BMP co-receptor a	[21]
TUBB2B	tubulin beta 2B class IIb	
LIFR	LIF receptor subunit alpha	[22]
LRRN2	leucine rich repeat neuronal 2	[23]
SPARCL1	SPARC like 1	[24]
ERBB2 (HER2)	erb-b2 receptor tyrosine kinase 2	[25]
MALAT1	metastasis associated lung adenocarcinoma transcript 1	
PCDH19	protocadherin 19	
HSPA6	heat shock protein family A (Hsp70) member 6	
HCAR2	hydroxycarboxylic acid receptor 2	
MYH7B	myosin heavy chain 7B	[26]
EPHB6	EPH receptor B6	[27]
MUC5AC	mucin 5AC, oligomeric mucus/gel-forming	[28]
CPS1	carbamoyl-phosphate synthase 1	[29]
ITLN2	intelectin 2	
RNU4-2	RNA, U4 small nuclear 2	

Among these 29 genes identified only after PSM are numerous well-known cancer-related markers, e.g., ERBB2, MALAT1, or MUC5AC. A literature search revealed that at least 15 of these markers have been suggested as potential diagnostic or predictive markers for CRC (see refences listed for each potential marker in Table 4). More extensive investigation of these markers and their roles in CRC will be warranted in future studies.

## Discussion

Propensity score matching (PSM) is a powerful statistical tool for controlling confounding factors in analyses of complex omics biomarker cohorts. In this study, we demonstrated a PSM strategy for creating two well-balanced groups that mimic the original study cohort but with significantly fewer cases or patients required while eliminating outcome-confounding clinical differences between the original cohorts. We created a stepwise matching protocol in which the cases were matched from the one with the lowest to the highest propensity score (Fig 1). This ascending sort should be better than a descending sort. As seen in our two datasets, cases with lower propensity scores are easier to match (Figs 2 and 4). If a descending sort were used, the paired case would consistently have a lower propensity score, and the covariates would be more likely to remain after matching. Therefore, we predicted that an ascending sort would result in a better matching outcome.

PSM is a cost-effective analytical method for omics data analyses and biomarker discovery. Obtaining large omics expression dataset is typically expensive, so cost is often a significant concern for research. By implementing PSM before the experimental process, high-quality case series can be created from a much smaller number of cases. This approach not only provides higher-quality analytical results but also lowers research costs. Additionally, PSM enables direct comparisons between patients. In past omics data analyses, the common method of comparison was between cancer and normal tissue to uncover cancer biomarkers. However, this method might not be appropriate for detecting certain cancer mechanisms, such as treatment response or drug resistance. PSM enables comparisons between patients, which can help identify these mechanisms directly.

In addition to the benefit of minimizing cohort size, PSM enabled us to discover several, potentially important markers for colorectal cancer. From the proteomic marker cohort, PSM affirmed the prognostic value of STAT1 in CRC that we have reported previously [30], and the conclusion was further independently solidified by random forest analysis in this study. From the CRC transcriptome cohort, PSM allowed us to identify several well-known prognostic factors that might have been missed in traditional differential expression analysis due to confounding factors, e.g., ERBB2 (HER2), MALAT1, and MUC5AC.

ERBB2 (also known as HER2, neu, and NGL) encodes a transmembrane glycoprotein belonging to the epidermal growth factor receptor family [31–33]. ERBB2 is one of the most extensively studied molecules in cancer research [34], particularly in breast cancer [35, 36]. Overexpression of ERBB2 in cancer cells has been linked to metastasis, poor response to cancer treatment, and poor prognosis. In our study, the mRNA abundance of ERBB2 was found to be significantly higher in the poor-prognosis group by post-PSM analysis. However, the significance of this difference was not identifiable without PSM.

MALAT1 (metastasis-associated lung adenocarcinoma transcript 1) is one of the first identified cancer-associated long noncoding RNAs. MALAT1 was originally described as a prognostic marker of lung cancer metastasis. Upregulated MALAT1 has been observed in various cancers, including CRC, and is associated with cancer metastasis, invasion, and poor prognosis [37–39]. In this study, despite previous reports linking upregulated MALAT1 to poor prognosis, we observed a significant downregulation of MALAT1 mRNA abundance in the poor prognosis group after PSM. It is worth noting that our study includes a high proportion of advanced-stage cases, with 35.7% of cases in TNM stage IV and 33.9% in stage III. Previous investigations into the expression level of MALAT1 have mainly focused on early-stage patients, and there are limited reports on the expression of MALAT1 in advanced stage patients.

MUC5AC is one of the mucins and a high molecular weight glycoprotein. While it is not expressed in normal colonic mucosa, it is expressed during CRC progression [40, 41]. However, the function of dysregulated MUC5AC expression has not been well characterized. A meta-analysis by Li et al. found that a high level of MUC5AC was associated with an improved prognosis [42]. Consistent with this, the mRNA abundance of MUC5AC was significantly decreased in the poor-prognosis group in our study. It is possible that MUC5AC plays a disease-modifying function in some CRC patients.

PSM also allowed us to discover several lesser studied or previously unknown biomarkers of CRC. For example, a MYH7B coding variant was discovered as one of the eight novel variants associated with CRC risk in a Swedish population from genome-wide association studies of 1,515 CRC patients and 12,108 controls [43]. MYH7B association with CRC risk was also reported in a gene expression prediction model of a large cohort of CRC cases that included 58,131 CRC cases and 67,347 controls of European ancestry [26]. By applying PSM, we identified the significance of MYH7B in CRC from a much smaller cohort than these two studies. Although further research is needed to confirm many of the potential new markers identified in our study, our results demonstrate the applicability and effectiveness of PSM for omics data analyses.

PSM analysis has some well-known limitations. It is crucial to list all possible confounding features without omission because the confounding factors that PSM can eliminate are only the ones that are already known. If unknown confounding features exist, the PSM analysis may not work correctly [44]. Additionally, PSM reduces the number of cases, and the result of post-PSM analysis may contain type 2 errors [45, 46]. In the differential expression analysis of RNA-seq dataset, this explains why there were significantly fewer genes in the analysis after PSM (122 genes after PSM vs. 402 genes before PSM). There is a possibility of overlooking some significant genes that should be focused on due to type 2 errors. This problem could be addressed by collecting a greater number of cases at the beginning. Using 2-to-1 matching in PSM can indeed be a useful arrangement to minimize the problem of type 2 errors [47, 48]. In this approach, two cases with most similar propensity scores are matched to one case in the opposite group. This can help to increase the number of cases while still maintaining balance in the covariates between the two groups. For the CRC proteomic marker dataset, we used this approach to match a good prognosis group of 104 cases to a poor prognosis group of 52 cases (S1 Fig, S2 and S3 Tables) with overall results very similar to 1-to-1 PSM. This arrangement can be useful in some datasets where increasing the number of cases is important while maintaining a balance of covariates.

## Conclusion

Propensity score matching (PSM) is a valuable and cost-effective method in cohort studies and omics data analyses as it allows researchers to create comparable groups with homogenized backgrounds using fewer cases. By reducing the impact of confounding factors, PSM can improve the accuracy and reliability of biomarker discovery.

## Supporting information

S1 Checklist Human participants research checklist. (DOCX)

S1 Fig Distribution of propensity scores of the IHC CRC dataset with a 2-to-1 matching arrangement. (TIF)

S1 Table The details of IHC antibody staining and scorings. (DOCX)

S2 Table Genes commonly selected before and after PSM. (DOCX)

S3 Table Clinicopathological features of IHC scoring dataset after PSM with a 2-to-1 matching arrangement. (DOCX)

S4 Table Comparison of IHC scoring between good and poor prognosis groups after 2-to-1 PSM. Bold: p<0.05.
(DOCX)

## List of abbreviations

PSM: Propensity score matching
CRC: Colorectal cancer
MSKCC: Memorial Sloan Kettering Cancer Center
TCGA: The cancer genome atlas
MMR: Mismatch repair
IHC: Immunohistochemical
CPM: Counts per million
FDR: False discovery rate
NNMT: Nicotinamide N-methyltransferase
GALNT6: Polypeptide N-acetylgalactosaminyltranferase 6
SLC7A5, SLC3A2: Solute carrier Family 7 member 5
SLC3A2: Solute carrier family 3 member
IGF2BP3: Insulin like growth factor 2 mRNA binding protein
MCM6: Minichromosome maintenance complex component 6
SERPIN B5: Serpin family B member 5
STAT1: Signal transducer and activator of transcription 1
NAMPT: Nicotinamide phosphoribosyltransferase
P4HA1: Prolyl 4-hydroxylase subunit alpha 1
LTBP2: Latent transforming growth factor beta binding protein 2
DDX21: DEAD-box RNA helicase 21
