# Supplementary figures and images for: Propensity score matching as an effective strategy for biomarker cohort design and omics data analysis
Source: PLoS One. 2024 May 2;19(5):e0302109. doi: 10.1371/journal.pone.0302109 (PMC11065211; doi:10.1371/journal.pone.0302109)

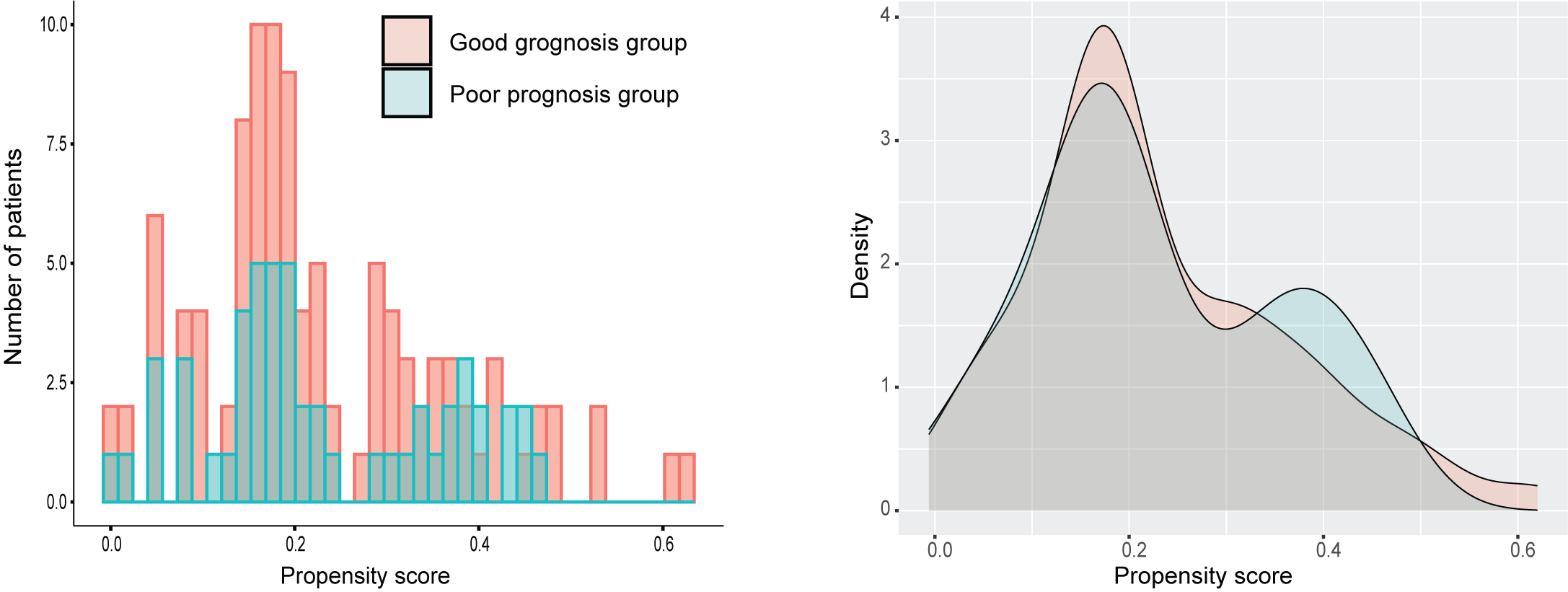

Supplement: S1 Fig — (TIF) [file pone.0302109.s002.tif]
