# Supplementary material for: Propensity score matching as an effective strategy for biomarker cohort design and omics data analysis
Source: PLoS One. 2024 May 2;19(5):e0302109. doi: 10.1371/journal.pone.0302109 (PMC11065211; doi:10.1371/journal.pone.0302109)
Supplement: S1 Table — (DOCX) [file pone.0302109.s003.docx]

| **Protein biomarker** | **Expression location** | **Specific antibody** | | | **IHC scoring** | |
| --- | --- | --- | --- | --- | --- | --- |
|  |  |  |  |  | Method | Range |
| NNMT | Stroma | HPA059180 | 1 : 200 | Atlas Antibodies, Sigma | Counting | 0-100% |
| GALNT6 | Cytoplasm | HPA011762 | 1 : 150 | Atlas Antibodies, Sigma | Counting | 0-100% |
| SLC3A2 | Membrane | HPA017980 | 1 : 4000 | Atlas Antibodies, Sigma | H-score | 0-300 |
| SLC7A5 | Membrane | HPA052673 | 1 : 50 | Atlas Antibodies, Sigma | H-score | 0-300 |
| IGF2BP3 | Cytoplasm | HPA002037 | 1 : 100 | Atlas Antibodies, Sigma | H-score | 0-300 |
| MCM6 | Nucleus | HPA004818 | 1 : 100 | Atlas Antibodies, Sigma | Counting | 0-100% |
| SERPIN B5 | Cytoplasm | HPA019025 | 1 : 2000 | Atlas Antibodies, Sigma | H-score | 0-300 |
| STAT1 | Cytoplasm | HPA000982 | 1 : 750 | Atlas Antibodies, Sigma | H-score | 0-300 |
| NAMPT | Cytoplasm | HPA047776 | 1 : 100 | Atlas Antibodies, Sigma | H-score | 0-300 |
| P4HA1 | Cytoplasm | HPA026593 | 1 : 2000 | Atlas Antibodies, Sigma | H-score | 0-300 |
| DDX21 | Nucleus | HPA036593 | 1 : 200 | Atlas Antibodies, Sigma | Positive / Negative | |
| LTBP2 | Cytoplasm | HPA003415 | 1 : 250 | Atlas Antibodies, Sigma | Positive / Negative | |
|  | Stroma |  |  |  | Positive / Negative | |
