# Supplementary material for: Propensity score matching as an effective strategy for biomarker cohort design and omics data analysis
Source: PLoS One. 2024 May 2;19(5):e0302109. doi: 10.1371/journal.pone.0302109 (PMC11065211; doi:10.1371/journal.pone.0302109)
Supplement: S2 Table — (DOCX) [file pone.0302109.s004.docx]

| Gene symbol | Description |
| --- | --- |
| TKTL1 | transketolase like 1 |
| FHL1 | four and a half LIM domains 1 |
| ATP2B4 | ATPase plasma membrane Ca2+ transporting 4 |
| PSD | pleckstrin and Sec7 domain containing |
| NTN1 | netrin 1 |
| MYLK | myosin light chain kinase |
| LIMS2 | LIM zinc finger domain containing 2 |
| SPEG | striated muscle enriched protein kinase |
| TACR2 | tachykinin receptor 2 |
| PPP1R12B | protein phosphatase 1 regulatory subunit 12B |
| TNS1 | tensin 1 |
| PTGS1 | prostaglandin-endoperoxide synthase 1 |
| SORBS1 | sorbin and SH3 domain containing 1 |
| MYL9 | myosin light chain 9 |
| MYOM1 | myomesin 1 |
| CALB1 | calbindin 1 |
| CLIP3 | CAP-Gly domain containing linker protein 3 |
| CAV1 | caveolin 1 |
| OGN | osteoglycin |
| NCS1 | neuronal calcium sensor 1 |
| C7 | complement C7 |
| NPR3 | natriuretic peptide receptor 3 |
| ANGPTL1 | angiopoietin like 1 |
| CASQ2 | calsequestrin 2 |
| NKX2-3 | NK2 homeobox 3 |
| CALD1 | caldesmon 1 |
| FLNC | filamin C |
| CNN1 | calponin 1 |
| KIF1A | kinesin family member 1A |
| AOC3 | amine oxidase copper containing 3 |
| RARA | retinoic acid receptor alpha |
| MYH11 | myosin heavy chain 11 |
| NIBAN1 | niban apoptosis regulator 1 |
| DNAJB5 | DnaJ heat shock protein family (Hsp40) member B5 |
| TGFB1I1 | transforming growth factor beta 1 induced transcript 1 |
| MYOCD | myocardin |
| GRB7 | growth factor receptor bound protein 7 |
| MIEN1 | migration and invasion enhancer 1 |
| AFF3 | ALF transcription elongation factor 3 |
| ANK2 | ankyrin 2 |
| KCNMB1 | potassium calcium-activated channel subfamily M regulatory beta subunit 1 |
| NCAM1 | neural cell adhesion molecule 1 |
| TAGLN | transgelin |
| JPH2 | junctophilin 2 |
| GFRA1 | GDNF family receptor alpha 1 |
| HSPB8 | heat shock protein family B (small) member 8 |
| SETBP1 | SET binding protein 1 |
| ABI3BP | ABI family member 3 binding protein |
| PGM5 | phosphoglucomutase 5 |
| PDLIM3 | PDZ and LIM domain 3 |
| KCNMA1 | potassium calcium-activated channel subfamily M alpha 1 |
| SLC30A2 | solute carrier family 30 member 2 |
| CSRP1 | cysteine and glycine rich protein 1 |
| ACTG2 | smooth muscle [Source:HGNC Symbol;Acc:HGNC:145] |
| LMOD1 | leiomodin 1 |
| PPBP | pro-platelet basic protein |
| SLC29A4 | solute carrier family 29 member 4 |
| CFL2 | cofilin 2 |
| RBPMS2 | mRNA processing factor 2 [Source:HGNC Symbol;Acc:HGNC:19098] |
| PPP1R14A | protein phosphatase 1 regulatory inhibitor subunit 14A |
| PSCA | prostate stem cell antigen |
| TNXB | tenascin XB |
| CAVIN2 | caveolae associated protein 2 |
| LINGO1 | leucine rich repeat and Ig domain containing 1 |
| TM4SF4 | transmembrane 4 L six family member 4 |
| NEGR1 | neuronal growth regulator 1 |
| SYNPO2 | synaptopodin 2 |
| EGFL7 | EGF like domain multiple 7 |
| ADCY5 | adenylate cyclase 5 |
| MSRB3 | methionine sulfoxide reductase B3 |
| DES | desmin |
| PRIMA1 | proline rich membrane anchor 1 |
| GNG7 | G protein subunit gamma 7 |
| TSPYL5 | TSPY like 5 |
| SYNM | synemin |
| SMTN | smoothelin |
| PDE2A | phosphodiesterase 2A |
| MAPT | microtubule associated protein tau |
| MAPK12 | mitogen-activated protein kinase 12 |
| PRELP | proline and arginine rich end leucine rich repeat protein |
| DAPK1 | death associated protein kinase 1 |
| FLNA | filamin A |
| KANK2 | KN motif and ankyrin repeat domains 2 |
| SVIL | supervillin |
| TPM2 | tropomyosin 2 |
| PLN | phospholamban |
| CCDC69 | coiled-coil domain containing 69 |
| NALF1 | NALCN channel auxiliary factor 1 |
| MT-RNR2 | mitochondrially encoded 16S rRNA |
| MTATP8P2 | MT-ATP8 pseudogene 2 |
| PEG10 | paternally expressed 10 |
| SUZ12P1 | SUZ12 pseudogene 1 |
| RN7SL1 | RNA component of signal recognition particle 7SL1 |
