# Supplementary material for: Propensity score matching as an effective strategy for biomarker cohort design and omics data analysis
Source: PLoS One. 2024 May 2;19(5):e0302109. doi: 10.1371/journal.pone.0302109 (PMC11065211; doi:10.1371/journal.pone.0302109)
Supplement: S3 Table — (DOCX) [file pone.0302109.s005.docx]

|  | **Good prognosis (N=104)** | **Poor prognosis (N=52)** | **p-value** |
| --- | --- | --- | --- |
| Age (mean) | 58.4 | 58.6 | 0.955 |
| Gender |  |  | 0.6828 |
| Male | 63 | 34 |  |
| Female | 41 | 18 |  |
| Location |  |  | 0.7735 |
| Right | 44 | 20 |  |
| Left | 60 | 32 |  |
| TNM staging (mean) | 2.20 | 2.21 | 0.9424 |
| Mismatch repair loss |  |  | 0.9385 |
| Present | 16 | 9 |  |
| Absent | 88 | 43 |  |
| Histology |  |  | 0.9184 |
| Mucinous | 8 | 5 |  |
| Not mucinous | 96 | 47 |  |
| Tumor differentiation (mean) | 2.07 | 2.06 | 0.8812 |
| Lympho-vascular invasion |  |  | 1 |
| Positive | 40 | 20 |  |
| Negative | 64 | 32 |  |
| Perineural invasion |  |  | 0.8139 |
| Positive | 17 | 7 |  |
| Negative | 87 | 45 |  |
