# Supplementary material for: Propensity score matching as an effective strategy for biomarker cohort design and omics data analysis
Source: PLoS One. 2024 May 2;19(5):e0302109. doi: 10.1371/journal.pone.0302109 (PMC11065211; doi:10.1371/journal.pone.0302109)
Supplement: S4 Table — Bold: p<0.05. (DOCX) [file pone.0302109.s006.docx]

|  | **Good prognosis (N=104)** | **Poor prognosis (N=52)** | **p-value** |
| --- | --- | --- | --- |
| NNMT | 39.4 | 37.7 | 0.5845 |
| GALNT6 | 53.1 | 52.9 | 0.9649 |
| SLC7A5 | 77.3 | 65.4 | 0.2203 |
| SLC3A2 | 67.8 | 69.1 | 0.9098 |
| IGF2BP3 | 104.8 | 95.6 | 0.3879 |
| MCM6 | 18.3 | 16.6 | 0.6885 |
| SERPIN B5 | 79.7 | 68.3 | 0.3025 |
| STAT1 | 56.3 | 23.3 | **< 0.001** |
| NAMPT | 96.6 | 89.2 | 0.4843 |
| P4HA1 | 112.5 | 106.3 | 0.5725 |
| LTBP2 (cytoplasm) positive | 36 | 23 | 0.321 |
| LTBP2 (stroma) positive | 25 | 11 | 0.8403 |
| DDX21 positive | 24 | 7 | 0.2278 |
